# Standard molar enthalpy of combustion and formation of quaternary ammonium tetrachlorozincate [*n*-C_n_H_2n+1_ N(CH_3_)_3_]_2_ ZnCl_4_

**DOI:** 10.1186/2193-1801-2-98

**Published:** 2013-03-09

**Authors:** Biyan Ren, Shuying Zhang, Bei Ruan, Kezhong Wu, Jianjun Zhang

**Affiliations:** 1 Department of Chemistry and Material Science, Hebei Normal University, Shijiazhuang, 050024 China; 2 Department of Basic Course, the Chinese People’s Armed Police Force Academy, Langfang, 065000 China

**Keywords:** Combustion calorimeter, Energy of combustion, Enthalpy of combustion, Enthalpy of formation, Quaternary ammonium tetrachlorozincate

## Abstract

The standard molar enthalpy of combustion (Δ_c_*H*^o^_m_) and formation (Δ_f_*H*^o^_m_) of quaternary ammonium tetrachlorozincate [*n*-C_n_H_2n+1_N(CH_3_)_3_]_2_ZnCl_4_ have been determined for the hydrocarbon chain length from even number 8 to 18 of carbon atoms (n) by an oxygen-bomb combustion calorimeter. The results indicated that the values of Δ_c_*H*^o^_m_ increased and Δ_f_*H*^o^_m_ decreased with increasing chain length and showed a linear dependence on the number of carbon atoms, which were caused by that the order and rigidity of the hydrocarbon chain decreased with increasing the carbon atoms. The linear regression equations are -Δ_c_*H*^o^_m_ =1440.50n +3730.67 and -Δ_f_*H*^o^_m_ = −85.32n + 1688.22.

## Introduction

The quaternary ammonium tetrachlorometallate with the general formula [*n-*C_n_H_2n+1_NR_3_]_2_MX_4_ (M = Cu, Mn, Cd, Zn, Co, …, X = Cl, Br, I, R is alkyl, or aryl) (short notation: C_n_C_3_M) have been attracted considerable attention because of their physical properties including ferro-, piezo- or pyroelectricity, ferri-, antiferro- or piezomagnetism and their technical application for electro- or magneto-optical devices (Blachnik et al. 1996; Kezhong et al. 2010). The advances in synthesis along with the ease of controlling various structural parameters (metal, halogen and number of carbon atoms in the alkylammonium ion) have made them ideal objects for studies by spectroscopy, calorimetry, diffraction, and a variety of other techniques (Abid et al. 2011; Donghua et al. 2011; Shymkiv et al. 2011). In addition, several theoretical studies have been undertaken to predict the behavior of the C_n_C_3_M (Francesco et al. 2002; Gosniowska et al. 2000). However, the thermodynamic properties of the C_n_C_3_M have been reported rarely in the literature. In the present work, the series of quaternary ammonium tetrachlorozincate [*n*-C_n_H_2n+1_ N(CH_3_)_3_]_2_ZnCl_4_ (n = 8, 10, 12, 14, 16, 18) are synthesized from ethanol solutions. The standard molar enthalpy of formation (Δ_f_*H*^o^_m_) and the standard molar enthalpy of combustion (Δ_c_*H*^o^_m_) of the C_n_C_3_Zn have been determined by an oxygen-bomb combustion calorimeter with increasing chain length at *T* = 298.15 K.

### Experimental procedure

ZnCl_2_, concentrated HCl and absolute ethanol were analytical grade. *n*-Octyltrimethylammonium chloride (A.P.), were purchased from TOKYO CHEMICAL INDUSTRY CO LTD (Japan). *n*-Decyltrimethylammonium chloride(A.P.), *n*-Dodecyltrimethylammonium chloride(A.P.), *n*-Tetradecyltrimethylammonium chloride(A.P.), *n*-Hexadecyltrimethylammonium chloride(A.P.), *n*-Trimethylstearylammonium chloride(A.P.) were purchased from J & K CHEMICAL LTD. For the synthesis of C_n_C_3_Zn, the hot absolute ethanol solutions of ZnCl_2_, concentrated HCl and the corresponding quaternary ammonium were mixed in a 1:2:2 molar ratios. The solutions were concentrated by boiling for 1 h, and then cooled to room temperature. After filtration, the products were recrystallized twice from absolute ethanol and then were placed in a vacuum desiccator for 10 h at about 353 K. The C_n_C_3_Zn were analyzed with an MT-3 CHN elemental analyzers (Japan) are listed in the following: Elemental analyses calc. (%) for C_8_C_3_Zn: C 47.88, H 9.43, N 5.08, Cl 25.75; Found: C 47.45, H 9.50, N 5.13, Cl 24.99. Anal. Calcd for C_10_C_3_Zn: C 51.37, H 9.88, N 4.61, Cl 23.38; Found: C 50.98, H 9.95, N 4.58, Cl 22.81. Anal. calcd for C_12_C_3_Zn: C 54.26, H 10.25, N 4.22, Cl 21.41; Found: C 53.93, H 10.34, N 4.26, Cl 21.25. Anal. Calcd for C_14_C_3_Zn: C 56.72, H 10.56, N 3.89, Cl 19.74; Found: C 56.06, H 10.20, N 3.84, Cl 19.03. Anal. Calcd for C_16_C_3_Zn: C 58.84, H 10.84, N 3.61, Cl 18.31; Found: C 58.91, H 10.77, N 3.62,Cl18.72. Anal. Calcd for C_18_C_3_Zn: C 60.65, H 11.07, N 3.37, Cl 17.08; Found: C 60.56, H 11.04, N 3.36, Cl 17.55.

The combustion experiments were performed with a static bomb calorimeter (XRY-1A Shanghai). Benzoic acid (Thermochemical Standard, BCS-CRM-190r) was used as calibrant of the bomb calorimeter. Its massic energy of combustion is *Δ*_*c*_*U* = −(26460 ± 3.8) J · g^−1^ under certificate conditions. The massic energy of combustion Δ_c_*U*_m_ for each C_n_C_3_Zn was fitted with equation Δ_c_*U*_m_ = [−*ε*_(calor)_ · Δ*T*+Δ*m*_*ign*_• *u*_*ign*_+*V*_NaOH_•(−59.7)]/*m*_CnC3Zn_, where *ε*_cal_ is the energy equivalent of the calorimeter, Δ*T* is the calorimeter temperature change corrected, Δ*m*_*ign*_ is the mass of the Nickel-chromium alloy for ignition and the massic energy is *u*_*ign*_ = −3.245 kJ · g^−1^(*U*_ign_ = Δ*m*_*ign*_• *u*_*ign*_). *m*_CnC3Zn_ is the mass of the C_n_C_3_Zn which were burned, *V*_NaOH_ is the volume of sodium hydroxid which consumed by nitric acid, the corrections for nitric acid formation were based on −59.7 kJ · mol^−1^ for the molar energy of formation of 0.1 mol · dm^−3^ HNO_3_ (aq) from N_2_, O_2_, and H_2_O(l) (Matos et al. 2002). The calibration results were corrected to the average mass of water added to the calorimeter: 2500.0 g and the volume of oxygen bomb was 300 ml. From five independent calibration experiments between *T* = 295.15 K and *T* = 299.15 K, the energy equivalent *ε*_cal_ = (13965.4 ± 4.7) J · K^-1^ was obtained, where the uncertainty quoted is the standard deviation of the mean. For all experiments, ignition was made at *T* = (298.150 ± 0.001) K. Combustion experiments were performed in oxygen at a pressure *p* = 3.00 MPa and in the presence of 10.00 cm^3^ of water added to the bomb (Matos et al. 2002).

## Results and discussion

The individual results of all combustion experiments, together with the mean values and their standard deviations, are given for each compound in Table 1. In accordance with normal thermochemical practice, the uncertainties assigned to the standard molar enthalpies of combustion are, in each case, equal to twice the overall standard deviation of the mean and include the uncertainties in calibration (Henoc et al. 2009). The results are referred to the following reactions (1 ~ 6) and the following equation (7 ~ 9):123456789

**Table 1:** **The values of the combustion energies of the quaternary ammonium tetrachlorometallate C**_**n**_**C**_**3**_**Zn**

	No.	***m***_CnC3Zn_/	Δ ***T*** /K	Δm_ign_/g	***U***_ign_/J	***V***_NaOH_/ml	***U***_NaOH_/J	-Δ_C_***U***_m_/kJ · g^−1^
C_8_C_3_Zn	1	0.4816	0.950	0.0033	10.71	3.88	23.20	27.478
	2	0.5028	0.990	0.0031	10.06	3.78	22.60	27.432
	3	0.4725	0.940	0.0041	13.30	4.08	24.40	27.703
	4	0.3775	0.750	0.0028	9.09	4.00	23.92	27.658
	5	0.5193	1.034	0.0025	8.11	4.40	26.31	27.741
	Ave.							27.602 ± 0.139
C_10_C_3_Zn	1	0.5081	1.085	0.0041	13.30	2.85	17.04	29.762
	2	0.5306	1.120	0.0039	12.66	2.89	17.28	29.422
	3	0.5035	1.070	0.0038	12.33	3.39	20.33	29.631
	4	0.4403	0.895	0.0030	9.74	2.30	13.75	29.804
	5	0.4630	0.950	0.0030	9.74	2.90	17.34	29.640
	Ave.							29.652 ± 0.149
C_12_C_3_Zn	1	0.4907	1.115	0.0047	15.25	3.03	18.12	31.666
	2	0.4591	1.040	0.0049	15.90	2.93	17.54	31.563
	3	0.4706	1.060	0.0044	14.28	3.25	19.44	31.385
	4	0.4566	1.040	0.0032	10.38	3.20	19.14	31.744
	5	0.4673	1.055	0.0040	12.98	3.55	21.23	31.455
	Ave.							31.563 ± 0.147
C_14_C_3_Zn	1	0.4699	1.120	0.0025	8.11	2.45	14.65	33.238
	2	0.5225	1.251	0.0037	12.01	3.65	21.83	33.372
	3	0.4130	0.985	0.0039	12.66	2.46	14.71	33.241
	4	0.5325	1.270	0.0047	15.25	2.45	14.65	33.251
	5	0.4362	1.040	0.0042	13.63	3.75	22.43	33.214
	Ave.							33.263 ± 0.062
C_16_C_3_Zn	1	0.5125	1.270	0.0046	14.93	2.50	14.97	34.549
	2	0.4720	1.160	0.0042	13.63	2.44	14.57	34.262
	3	0.4902	1.210	0.0027	8.76	3.04	18.18	34.417
	4	0.4758	1.170	0.0043	13.95	3.01	17.99	34.274
	5	0.4577	1.130	0.0044	14.28	3.94	23.56	34.396
	Ave.							34.379 ± 0.118
C_18_C_3_Zn	1	0.5605	1.430	0.0030	9.74	3.04	18.18	35.580
	2	0.5755	1.475	0.0058	18.82	3.03	18.12	35.729
	3	0.5283	1.348	0.0062	20.12	3.42	20.45	35.557
	4	0.4465	1.140	0.0034	11.03	3.65	21.83	35.583
	5	0.4459	1.130	0.0038	12.33	3.65	21.83	35.315
	Ave.							35.552 ± 0.149

Where *R* is the molar gas constant and M is the molar mass of the C_n_C_3_Zn. The V_B_ is the stoichiometric coefficient and the Δ_f_*H*^o^_m_ (B) is the standard molar enthalpy of formation of the combustion products. The standard molar enthalpies of formation of ZnO(s), H_2_O (l) and CO_2_(g) at *T* = 298.15 K, −(348.28) kJ · mol^−1^, −(285.830 ± 0.042) kJ · mol^−1^ and − (393.51 ± 0.13) kJ · mol^−1^ (Manuel et al. 2010). The Δ_f_*H*^o^_m_ of the C_n_C_3_Zn resulted from the Δ_c_*H*^o^_m_ by an oxygen-bomb combustion calorimeter at *T* = 298.15 K. Table 2 lists the values of the standard molar energies Δ_c_*U*^o^_m_, the enthalpies of combustion Δ_c_*H*^o^_m_ and the standard molar enthalpies of formation Δ_f_*H*^o^_m_ result form Δ_c_*U*^o^_m_ for the C_n_C_3_Zn.

**Table 2:** **The value of thermochemical functions of the quaternary ammonium tetrachlorometallate C**_**n**_**C**_**3**_**Zn**

	C_8_C_3_Zn	C_10_C_3_Zn	C_12_C_3_Zn	C_14_C_3_Zn	C_16_C_3_Zn	C_18_C_3_Zn
-Δ_c_*U*^o^_m_/kJ · mol^-1^	15268	18010	20938	23929	26657	29557
-Δ_c_*H*^o^_m_/kJ · mol^−1^	15297	18044	21009	24006	26740	29647
-Δ_f_*H*^o^_m_/kJ · mol^−1^	991.66	908.02	660.38	380.47	364.10	174.46

**Figure 1:**
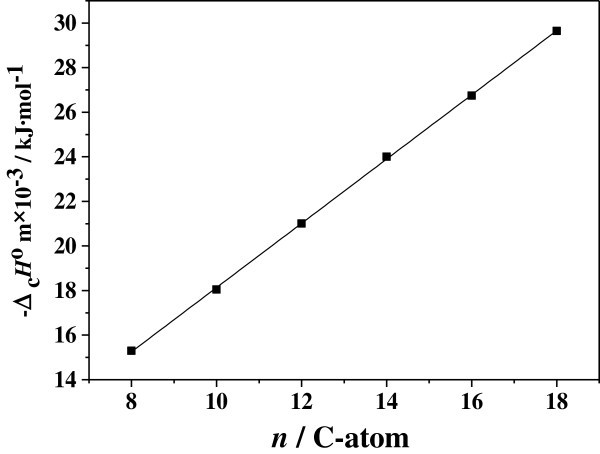
**Plot of Δ**_**c**_***H***^**o**^_**m**_**vs. number(*n*) of carbon-atoms in the quaternary ammonium tetrachlorometallate C**_***n***_**C**_**3**_**Zn.**

**Figure 2:**
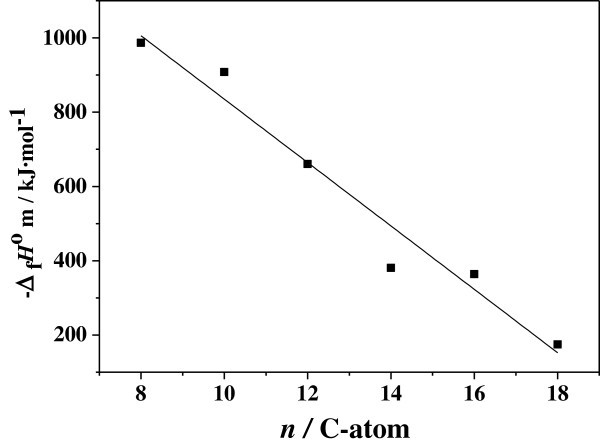
**Plot of Δ**_**f**_***H***^**o**^_**m**_**vs. number(*n*) of carbon-atoms in the quaternary ammonium tetrachlorometallate C**_***n***_**C**_**3**_**Zn.**

The influence of the hydrocarbon chain length on Δ_c_*H*^o^_m_ and Δ_f_*H*^o^_m_ of the C_n_C_3_Zn has been obtained for chain lengths from 8 to 18 carbon atoms. The values of Δ_c_*H*^o^_m_ and Δ_f_*H*^o^_m_ show a linear dependence on the number of carbon atoms from experimental data analysis. Figure 1, Figure 2 show a plot of the calculated -Δ_c_*H*^o^_m_ and -Δ_f_*H*^o^_m_ vs. C-atoms (n) that gave a straight line relationship from the values of Table 2. The linear regression equation are -Δ_c_*H*^o^_m_ =1440.50n +3730.67 with a correlation coefficient *r* = 0.9998 and - Δ_f_*H*^o^_m_ = −85.32n + 1688.22 with *r* =0.9512. A striking feature is that Δ_c_*H*^o^_m_ increased and Δ_f_*H*^o^_m_ decreased with increasing the chain length. This reason is that the structures of C_n_C_3_Zn are characteristic of the piling of sandwiches in which a two-dimensional cavities of ZnCl_4_^2-^ tetrahedra is sandwiched between two alkylammonium layers. The layers are bound by van der Waals forces between (CH_2_)_n_CH_3_ groups and by long-range Coulomb forces. The –N(CH)_3_^3+^ groups of the chains occupy the cavities of the ZnCl_4_^2-^ layers and are bonded by ion bonds to the chlorine atoms (Weizhen et al. 2011). As the hydrocarbon chain length increases, the formation of the chain conformer plays a more important role in the structural phase transitions. It is known that the order and rigidity of the hydrocarbon chain were decreased with increasing the carbon atoms, that is with increasing mean number of conformationally flexible chain in C_n_C_3_Zn (Nobuaki et al. 2011), furthermore, the intensities of the ion bonds and van der Walls force decrease with increasing the carbon atoms resulting in that the values of Δ_c_*H*^o^_m_ and Δ_f_*H*^o^_m_ show a linear dependence on the carbon atoms.

## Conclusions

The standard molar enthalpy of combustion and formation of quaternary ammonium tetrachlorozincate [*n*-C_n_H_2n+1_ N(CH_3_)_3_]_2_ZnCl_4_ (n = 8, 10, 12, 14, 16, 18) have been measured by an oxygen-bomb combustion calorimeter. The results indicated that the values of the standard molar combustion enthalpies Δ_c_*H*^o^_m_ of these compounds increased with increasing chain length and the standard molar formation enthalpies Δ_f_*H*^o^_m_ of these compounds decreased with increasing chain length and showed a linear dependence on the number of carbon atoms.
